# Circulating Tumor Reactive KIR+CD8+ T cells Suppress Anti-Tumor Immunity in Patients with Melanoma

**DOI:** 10.21203/rs.3.rs-3956671/v1

**Published:** 2024-02-28

**Authors:** David Hafler, Benjamin Lu, Liliana Lucca, Wesley Lewis, Jiping Wang, Catarina Nogeuira, Sebastian Heer, Pierre-Paul Axisa, Nicholas Buitrago-Pocasangre, Giang Pham, Mina Kojima, Wei Wei, Lilach Aizenbud, Antonietta Bacchiocchi, Lin Zhang, Joseph Walewski, Veronica Chiang, Kelly Olino, James Clune, Ruth Halaban, Yuval Kluger, Anthony Coyle, Jan Kisielow, Franz-Josef Obermair, Harriet Kluger

**Affiliations:** Yale University; Yale School of Medicine; Yale School of Medicine; Yale University; Yale School of Public Health; Repertoire Immune Medicines; Repertoire Immune Medicines; Yale University; Yale Medical School; Yale School of Medicine; Department of Genetics, Yale University; Yale School of Medicine; Yale School of Medicine; Yale School of Medicine; Yale School of Medicine; Yale School of Medicine; Yale School of Medicine; Yale School of Medicine; Yale School of Medicine; Yale University School of Medicine; Yale University School of Medicine; Repertoire Immune Medicines; Repertoire Immune Medicine (Switzerland); Repertoire Immune Medicine (Switzerland); Yale University School of Medicine

## Abstract

Effective anti-tumor immunity is largely driven by cytotoxic CD8^+^ T cells that can specifically recognize tumor antigens. However, the factors which ultimately dictate successful tumor rejection remain poorly understood. Here we identify a subpopulation of CD8^+^ T cells which are tumor antigen-specific in patients with melanoma but resemble KIR^+^CD8^+^ T cells with a regulatory function (Tregs). These tumor antigen-specific KIR^+^CD8^+^ T cells are detectable in both the tumor and the blood, and higher levels of this population are associated with worse overall survival. Our findings therefore suggest that KIR^+^CD8^+^ Tregs are tumor antigen-specific but uniquely suppress anti-tumor immunity in patients with melanoma.

## Introduction

Over the past decade, therapies modulating the immune system’s ability to identify and lyse cancer cells have revolutionized cancer care. Monoclonal antibodies targeting negative signaling receptors on immune cells - termed checkpoint inhibitors - to break tolerance on functionally exhausted tumor antigen-specific T cells have improved survival across multiple cancer types, as exemplified by the durable clinical responses seen in > 50% of patients with advanced melanoma treated with combination anti-PD-1 and anti-CTLA-4 therapy^1^. However, the cellular and environmental elements dictating the success of anti-tumor immunity are complex and remain poorly understood, which limits the development of novel therapies. The presence of certain cell types, such as tumor antigen-specific CD8^+^ T cells and CD4^+^ regulatory T cells, are critical cellular elements to sustain or impair anti-tumor immune responses, respectively. However, how features of tumor antigen-specific T cells relate to circulating T cells and the clinical relevance of either population remain poorly characterized.

Here, we used single-cell RNA sequencing paired with T cell receptor (TCR) sequencing, multi-parametric flow cytometry, and high-throughput peptide screens to characterize tumor antigen-specific T cells in resected tumors and blood from patients with melanoma. Combining a transcriptional-based approach to predict tumor antigen reactivity with experimental confirmation of antigen specificity, we identify a subpopulation of tumor antigen-specific CD8^+^ T cells which are shared between tumor and blood and resemble KIR^+^CD8^+^ regulatory T cells (Tregs) recently described to have a role in regulating autoinflammatory responses^2,3^. Moreover, the frequencies of circulating KIR^+^CD8^+^ Tregs in patients with melanoma correlate with worse overall survival. These results provide new evidence for a role of CD8^+^ Tregs in regulating human immune responses that may have clinical importance as an alternative mechanism of tumor immune evasion.

## Results

### Circulating tumor antigen reactive T cells are predominantly clonally expanded, cytotoxic CD8^+^ T cells

**Figure 1:**
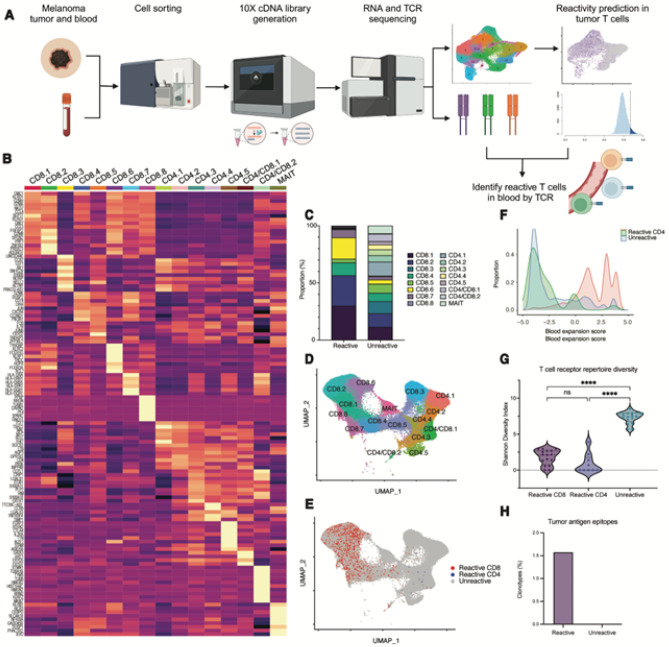
Circulating tumor antigen reactive T cells in patients with advanced melanoma. A) Design and analytical pipeline of 5’ droplet-based single-cell RNA sequencing with paired T cell receptor sequencing experiment to identify tumor antigen reactive T cells. B) Heatmap displaying the top ten differentially expressed genes of T cell transcriptional clusters identified from single-cell RNA sequencing. C) Proportion of transcriptional clusters in tumor antigen predicted-reactive or predicted-unreactive T cells. D) UMAP dimensionality reduction plot of circulating T cells labeled with transcriptional clusters or E) predicted tumor antigen reactivity. F) Histogram of the log-normalized proportion of the total T cell repertoire occupied by a given clonotype (“Expansion Score”) based on predicted tumor antigen reactivity. G) Violin plot of the T cell receptor repertoire diversity for each predicted reactivity group, as quantified by the Shannon Diversity Index. Diversity metrics for each patient are represented by separate dots. Between group differences are measured by Kruskal-Wallis one-way analysis of variance (*p* < 0.05, **** *p* < 0.001).

To understand the transcriptional and clonal relatedness of tumor antigen reactive T cells in the tumor and blood, we performed droplet-based, single-cell RNA sequencing with paired TCR sequencing in sorted immune cells from resected tumors and blood of 17 patients with immunotherapy-naive, cutaneous melanoma (Fig. 1A, **table S1**). In total, 78,840 tumor and 124,329 blood T cells were analyzed, of which 42,094 (53.4%) and 66,114 (53.2%) had paired TCR α- and β-chains in the tumor and blood, respectively. We used a stringent definition for clonal relatedness, whereby clonally related T cells were defined as those with identical single CDR3α- and single CDR3β-chain pairs based on amino acid sequences.

Using previously described, functionally validated gene signatures (neoTCR8 and neoTCR4 for CD8^+^ and CD4^+^ T cells, respectively)^4^, we then performed single-cell gene set enrichment analysis to predict the tumor antigen reactivity of T cells in the tumor (predicted-reactive T cells). We validated the accuracy of this transcriptional-based classification using external datasets annotated with experimentally-observed tumor antigen reactivity (**fig. S1**)^5,6^. Using TCR sequences to link clonally related T cells, we identified 7,183 CD8^+^ T cells (17.8% of total CD8^+^) and 145 CD4^+^ T cells (0.71 % of total CD4^+^) in the blood which were clonally related to predicted-reactive T cells in the tumor (Fig. 1B). Consistent with prior work, we noted a wide range in the frequency of circulating predicted-reactive T cells across patients (CD8^+^ T cells 0–50.6%, CD4^+^ T cells 0–4.28%) (**fig. S2**)^4,7–11^. We additionally identified 16,387 circulating T cells clonally related to predicted-unreactive tumor T cells.

Unsupervised clustering analysis of circulating αβ T cells revealed 16 distinct transcriptional clusters, including eight clusters composed primarily of CD8^+^ T cells, five clusters of CD4^+^ T cells, two clusters with mixed CD4^+^ and CD8^+^ T cells, and one cluster of mucosal-associated invariant T cells (Fig. 1B–E, **table S4**). Consistent with prior work, circulating predicted-reactive CD8^+^ T cells were concentrated in clusters with high cytotoxicity (CD8.1, CD8.2, CD8.4), high NK-associated markers (CD8.6), and which largely resembled terminally differentiated effector CD8^+^ T cells based on reference mapping (**fig. S3**), although they could be detected across nearly all transcriptional CD8^+^ clusters (Fig. 1C)^7,8,10,12^. In contrast, circulating CD4^+^ T cells were largely concentrated in clusters highly expressing *PASK, IL7R*, and *SELL* (CD4.3, CD4/CD8.1), consistent with central/effector memory-like phenotypes. These findings suggest that a continuum of transcriptional states in predicted-reactive CD8^+^ T cells may be captured within the blood.

To quantify the degree of clonal expansion, we calculated the proportion of the total TCR repertoire occupied by each clonotype (Expansion Score) and observed that predicted-reactive CD8^+^ T cells in the blood were more largely expanded than predicted-unreactive T cells (Fig. 1F). Consistent with a higher degree of clonal expansion, circulating predicted-reactive T cells also had more restricted TCR repertoires (Fig. 1G, **fig. S4**). Using a publicly available database of TCR sequences annotated with antigen reactivity (VDJdb^13^), we observed that 1.5% of predicted-reactive and no predicted-unreactive clonotypes in the blood matched TCR αβ amino acid sequences previously reported to be tumor antigen-specific (Fig. 1H). Our findings are thereby consistent with prior work showing that tumor antigen-specific T cells in the blood are predominantly a clonally expanded, cytotoxic CD8^+^ T cell population^7,10–12,14^.

### A subpopulation of circulating, predicted-reactive CD8^+^ T cells resemble KIR^+^CD8^+^ Tregs

**Figure 2:**
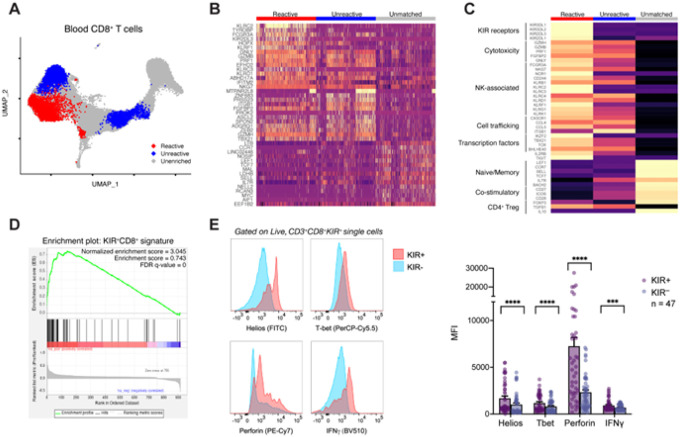
KIR^+^CD8^+^ regulatory T cells are differentially abundant in circulating tumor antigen reactive CD8^+^ T cells. A) UMAP dimensionality reduction plot of circulating CD8^+^ T cells labeled with differentially abundant predicted-reactive (red) and predicted-unreactive (blue) populations using Local Two-Sample Testing. B) Heatmap displaying the top 15 differentially expressed genes per differential abundance cluster. C) Heatmap displaying the expression of hallmark genes of human KIR^+^CD8^+^ regulatory T cells in each differentially abundant CD8^+^ T cell group. D) Gene set enrichment analysis of the top 200 upregulated genes from human KIR^+^CD8^+^ regulatory T cells from patients with multiple sclerosis in the differentially abundant, circulating tumor antigen reactive CD8^+^ T cells. E) Representative histograms (left) of intracellular staining patterns of Helios, T-bet, perforin, or interferon gamma in circulating KIR^+^CD8^+^ or KIR^−^CD8^+^ T cells from patients with advanced melanoma (n = 47). Bar graph summary of the geometric mean fluorescence intensity in KIR^+^CD8^+^ or KIR^−^CD8^+^ T cells. Between group differences are measured by Wilcoxon matched-pairs signed rank testing (*p* < 0.05, **** *p* < 0.001).

To derive a transcriptional signature with greater specificity for predicted-reactive T cells in the blood, we focused our analyses on CD8^+^ T cells which could be found in both the tumor and blood (tumor matched). We performed local two-sample testing by random-walk distributions to identify subpopulations of cells differentially abundant for tumor matched, predicted-reactive or predicted-unreactive CD8^+^ T cells (Fig. 2A)^15^. We identified a subpopulation of 3,051 cells enriched for predicted-reactive CD8^+^ T cells. Consistent with previous studies^7^, circulating CD8^+^ T cells clonally related to tumor-infiltrating T cells had high expression of genes related to cytotoxicity (*GNLY, GZMB, FGFBP2*) and cell migration (*ITGB1*) regardless of predicted reactivity (Fig. 2B, **table S5–6**). However, the subpopulation of predicted-reactive CD8^+^ T cells had high expression of several natural killer cell-associated genes (*NKG7, KLRD1, KLRC2, KLRC3, FCG3A*), including genes encoding the inhibitory subset of killer cell immunoglobulin-like receptor (KIR) family (e.g. *KIR2DL3*). While KIR expression is known to be upregulated in terminally differentiated CD8^+^ T cells^16,17^, human KIR^+^CD8^+^ T cells were also recently demonstrated to have regulatory-like function (KIR^+^CD8^+^ Tregs) in the context of autoimmune diseases and viral infections^2,18,19^. Similar to the better characterized CD4^+^ Tregs, KIR^+^CD8^+^ Tregs limit the effector function of pro-inflammatory immune cells, though do so through contact-dependent cytolysis^2^. Previous studies have also reported the presence of KIR^+^CD8^+^ Tregs in the context of tumor immune surveillance^3,20^, though the antigen specificity and clinical significance remain unclear.

To interrogate whether the identified subpopulation of circulating predicted-reactive KIR^+^CD8^+^ T cells may represent KIR^+^CD8^+^ Tregs, we examined the expression pattern of several hallmark genes (Fig. 2C). In line with KIR^+^CD8^+^ Tregs in patients with autoimmune diseases, the predicted-reactive KIR^+^CD8^+^ T cell population we observed highly expressed genes encoding several members of inhibitory KIRs (*KIR2DL1, KIR2DL3, KIR3DL1, KIR3DL2*) in addition to cytotoxic molecules (*GZMB, PRF1, GNLY*) and cell trafficking proteins (*CX3CR1, CCL4, CCL5, ITGB1*) hypothesized to be involved in their regulatory function. Similar to previously described CD8^+^ Tregs, the predicted-reactive KIR^+^CD8^+^ T cells lacked expression of co-stimulatory receptors (*CD27, CD28, ICOS*) and lacked expression of the CD4^+^ Treg-defining transcription factor *FOXP3*^2,21^. There was also high expression of *IKZF2*, which encodes the transcription factor Helios highly expressed by KIR^+^CD8^+^ Tregs in humans and is an essential element for the suppressive function of Tregs, including the analogous Ly49^+^CD8^+^ Treg population in mice^2,22–24^. Notably, the aggregate pattern of expression was only present in the subpopulation of predicted-reactive KIR^+^CD8^+^ T cells in comparison with other transcriptional clusters of blood CD8^+^ T cells (**fig. S5**). Gene set enrichment analysis also showed significant enrichment of the top 200 upregulated genes in KIR^+^CD8^+^ Tregs from patients with multiple sclerosis (Fig. 2D; normalized enrichment score = 3.045, false discovery rate = 0)^2^.

To confirm the protein-level expression of hallmark genes, we performed multi-parametric flow cytometry on peripheral blood mononuclear cells from an additional 47 patients with advanced melanoma (Fig. 2E, **fig. S6, table S10**). Relative to KIR^−^CD8^+^ T cells, KIR^+^CD8^+^ T cells had a higher protein-level expression of the transcription factors Helios (geometric mean fluorescence intensity (gMFI) 1,685 ± 240 vs. 1,013 ± 172; *p* < 0.001) and T-bet (gMFI 1,183 ± 133 vs. 807 ± 97.5; *p* < 0.001) in addition to the cytolytic protein perforin (gMFI 7,250 ± 943 vs. 2,322 ± 268; *p* < 0.001) and the effector cytokine interferon gamma (gMFI 926 ± 90 vs. 686 ± 47; *p* < 0.001), which are consistent with our transcriptional findings. These data suggest transcriptional and protein-level similarities between a subpopulation of predicted-reactive CD8^+^ T cells and previously described KIR^+^CD8^+^ Tregs.

### Hallmark transcriptional features of KIR^+^CD8^+^ Tregs are conserved in blood and tumor

**Figure 3:**
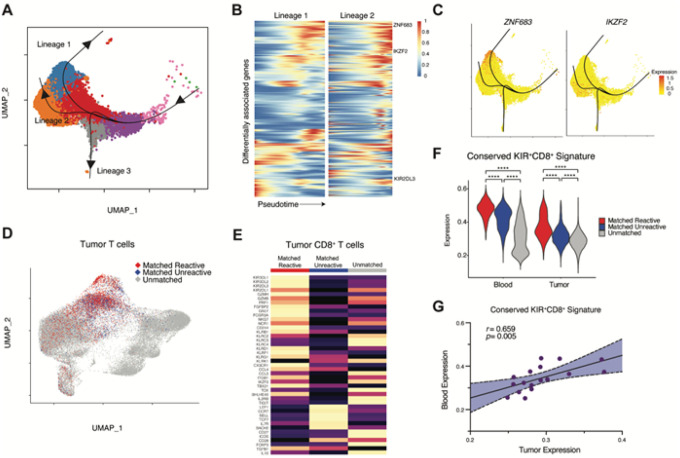
Relationship between circulating and tumor KIR^+^CD8^+^ tumor antigen reactive T cells. A) UMAP dimensionality reduction plot of circulating KIR^+^CD8^+^ tumor antigen reactive T cells and all clonally related cells with superimposed pseudotime trajectory curves. Naive-like T cells were defined as the pseudotime start point (right side of the plot). KIR^+^CD8^+^ reactive T cells are enriched along lineage 2. B) Diffusion heatmap displaying differentially associated genes along pseudotime (x-axis) in lineage 1 (left) as compared with lineage 2 (right). C) Gene expression of *ZNF683* (left, encodes the transcription factor Hobit) and *IKZF2* (right, encodes the transcription factor Helios) displayed on UMAP plots. D) UMAP dimensionality reduction plot of tumor T cells labeled with clonal relationship with circulating, differentially abundant predicted-reactive or predicted-unreactive CD8^+^ T cells. E) Heatmap of the expression of hallmark KIR^+^CD8^+^ regulatory T cell genes in tumor T cells. F) Violin plots summarizing the conserved KIR^+^CD8^+^ T cell gene signature. Between group differences are measured by Wilcoxon signed-rank testing with continuity correction (*q* < 0.05, **** *p* < 0.001). G) The correlation between the conserved KIR^+^CD8^+^ T cell gene signature in tumor (x-axis) or blood (y-axis) CD8^+^ T cells.

To understand how the differentiation state of predicted-reactive KIR^+^CD8^+^ T cells relates to other CD8^+^ T cells in the blood, we performed pseudotime trajectory analysis on the subpopulation of predicted-reactive T cells and clonally related cells. Defining clonally related cells with a naive differentiation state as the start of the differentiation trajectory, we observed three branched lineages (Fig. 3A). The subpopulation of predicted-reactive KIR^+^CD8^+^ T cells were enriched along lineage 2 while cells transcriptionally resembling predicted-unreactive CD8^+^ T cells were enriched in lineage 1. Lineage 3 was enriched with a minor population of proliferative cells.

To understand which genes are most associated with differentiation across lineages, we calculated the association between gene expression and pseudotime along each trajectory then identified genes which were differentially associated between lineages (Fig. 3B, **table S7**). *IKZF2* was among the most differentially associated genes along lineage 2 in comparison with other lineages (Wald statistic = 622.9, *p* = 0), which is consistent with the hypothesized role in defining the suppressive function of KIR^+^CD8^+^ Tregs (Fig. 3C). In comparison, *ZNF683*, which encodes the transcription factor Hobit, instructs tissue residency, and regulates cytotoxicity in lymphocytes^25–28^, was the most highly differentially associated gene in lineage 1 (Wald statistic = 3184.8, *p* = 0).

To understand whether the transcriptional state of predicted-reactive KIR^+^CD8^+^ T cells was also present in the tumor microenvironment, we examined the gene expression profile of 3,037 clonally related cells in the tumor (Fig. 3D). We again observed a high expression of hallmark KIR^+^CD8^+^ Treg genes and an absence of *FOXP3* (Fig. 3E). As inhibitory KIR proteins are known to be expressed in terminally differentiated CD8^+^ T cells and contain a cytoplasmic immunoreceptor tyrosine-based inhibitory motif (ITIM) domain, we examined whether KIR^+^CD8^+^ T cells in the tumor expressed features of T cell exhaustion (*PDCD1, HAVCR2, LAG3, TIGIT, CTLA4, ENTPD1, ITGAE, CXCL13, LAYN, CXCL13, IFNG*). Compared with other tumor T cells, KIR^+^CD8^+^ T cells had lower exhaustion gene module scores (*p* < *0.0001* vs. Other neoTCR8 reactive, *p* < 0.0001 vs. Unreactive; **fig. S7**), whereas other neoTCR8 predicted-reactive T cells had the highest features of exhaustion. These data suggest that CD8^+^ T cells that are clonally related to circulating predicted-reactive KIR^+^CD8^+^ T cells share hallmark features of KIR^+^CD8^+^ Tregs and lack features of T cell exhaustion induced by chronic antigen stimulation.

We next identified genes that distinguish the subpopulation of predicted-reactive KIR^+^CD8^+^ T cells in the blood and clonally related T cells in the tumor from all other CD8^+^ T cells in the blood and tumor, respectively. Genes that were differentially expressed by both predicted-reactive KIR^+^CD8^+^ T cells in the blood and clonally related cells in the tumor were considered to have conserved expression across tissue compartments (Fig. 3F; **table S8**). The conserved gene signature was comprised of 135 genes and again included genes encoding the KIR protein (*KIR2DL3*), cytotoxic molecules (*GNLY, PRF1, GZMB*), transcription factors (*TBX21, IKZF2*), NK cell-associated proteins (*KLRC2, NKG7, KLRD1, KLRG1*), and cell trafficking molecules (*CX3CR1, ITGB2, S1PR5*) in addition to the immunosuppressive cytokine transforming growth factor-β (*TGFB1*). Using the conserved gene signature, we next calculated gene expression module scores in all CD8^+^ T cells in the tumor and blood. Expression of the conserved signature in the tumor were positively correlated with those in the blood (*R*^2^ = 0.394, *p* = 0.005; Fig. 3G). In aggregate, these data suggest that KIR^+^CD8^+^ T cells represent a distinct differentiation state, and that the core transcriptional program is preserved across tissue compartments. Furthermore, the conserved gene signature in the blood is correlated with the expression in the tumor, providing support for using blood sampling to assess the presence of this population in the tumor.

### Functional confirmation of tumor antigen reactivity in KIR^+^CD8^+^ T cells

**Figure 4:**
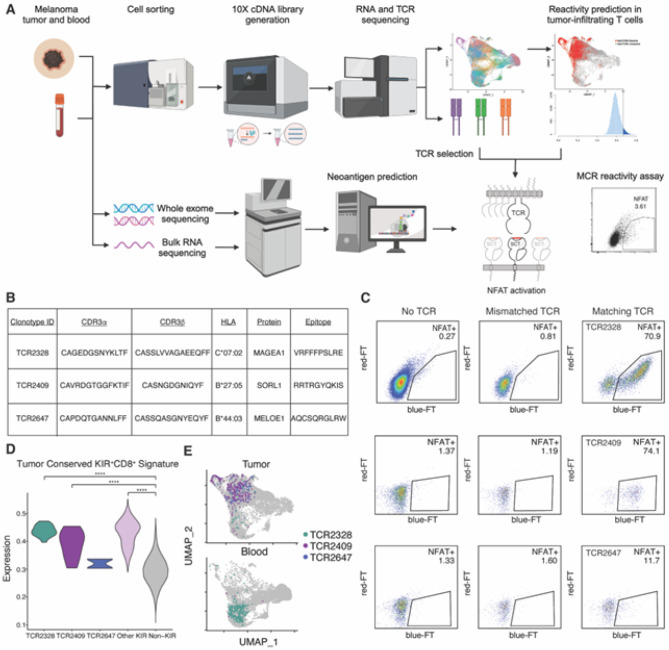
High-throughput epitope screening demonstrates tumor antigen specificity in KIR^+^CD8^+^ T cell clonotypes. A) Experimental design of epitope screening platform to identify TCR specificity using ten amino acid peptide sequences (10-mer) and major histocompatibility complex hybrid molecules (MCR). B) Table summarizing KIR^+^CD8^+^ T cell clonotype specificity from the MCR screen. C) Flow cytometry plots of responding MCR reporter cells (NFAT^+^) when co-cultured with 16.A2 cells carrying T cell receptors derived from KIR^+^CD8^+^ T cells or controls. D) Conserved KIR^+^CD8^+^ gene signature expression in tumor CD8^+^ T cells, grouped by KIR^+^CD8^+^ T cell clonotypes with demonstrated MCR reactivity (TCR2328, TCR2409, TCR2647), other KIR^+^CD8^+^ T cells, or KIR^−^CD8^+^ T cells. Between group differences are measured by Wilcoxon matched-pairs signed rank testing (*p* < 0.05, **** *p* < 0.001). E) UMAP dimensionality reduction plot of all CD8^+^ T cells split by the tissue of origin with KIR^+^CD8^+^ T cell clonotypes with demonstrated MCR reactivity (TCR2328, TCR2409, TCR2647) highlighted.

To functionally confirm the tumor antigen specificity of predicted-reactive KIR^+^CD8^+^ T cells, we performed an unbiased epitope screen in 12 patients with melanoma for whom we have previously performed paired single-cell RNA and T cell receptor sequencing (Fig. 4A, **table S1**)^7^. We selected candidate TCRs from a broad representation of transcriptional and clonal expansion states, including T cells with high expression of the neoTCR8 and the conserved KIR^+^CD8^+^ gene signatures, which were then cloned into a T cell line. Epitope screening was performed by selecting peptides related to known tumor associated antigens, melanoma driver mutations, human endogenous retroviral elements, and non-tumor protein as controls. For six patients, we also included personalized tumor neoantigens identified through whole-exome sequencing data of tumor from the same patient. Ten amino acid peptide sequences (10-mer) were cloned into single-chain trimers linked to an intracellular domain of TCR zeta chain (SCTz) to construct peptide and major histocompatibility complex hybrid molecules (MCR)^29,30^. In total, over 60,000 10-mers were included in the MCR library. The MCR were then transfected into 16.2X cell line with an NFAT-low fluorescent timer (sFT) reporter and co-cultured with the T cell line expressing selected TCRs.

Of 96 KIR^+^CD8^+^ clonotypes screened, three clonotypes (TCR2328, TCR2409, TCR2647) from separate patients were confirmed to be reactive to tumor antigens (Fig. 4B–E). TCR2328 and TCR2647 were reactive to known tumor associated antigens (MAGEA1^31^ and MELOE-1^32^, respectively), while TCR2409 was reactive to a neoantigen derived from a personalized mutation in the *SORL1* gene. Screened clonotypes from KIR^−^CD8^+^ T cells that were specific to tumor antigens were all predicted to be reactive based on neoTCR8 signatures and are the subject of further studies (manuscript under preparation). Notably, of all clonotypes specific to tumor antigens, two of the three KIR^+^CD8^+^ clonotypes (TCR2328, TCR2409) were the only TCRs identified in both tumor and blood in the single-cell sequencing data (Fig. 4E). While the demonstrated reactivity was limited to a minority of clonotypes, which is in line with work by others^5,6,10,29,30,33–35^ these data suggest that at least a subpopulation of KIR^+^CD8^+^ T cells are tumor antigen-specific.

### Tumor antigen reactive KIR^+^CD8^+^ T cells are associated with worse clinical outcome

**Figure 5:**
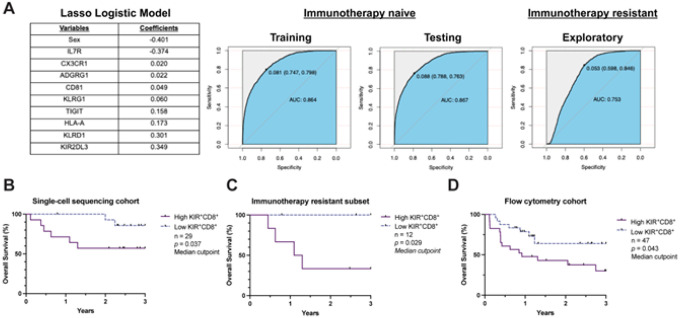
Clinical relevance of tumor antigen reactive, KIR^+^CD8^+^ T cells. A) Table of clinical and surface protein-encoding genes selected by the Lasso Logistic model to classify circulating CD8^+^ T cells as tumor antigen reactive, KIR^+^CD8^+^ T cells (left). Receiver-operating curves summarizing the predictive accuracy of the Lasso Logistic model classifier in the immunotherapy-naive (center) and immunotherapy-resistant cohorts (right). B) Three-year overall survival based on a high (> median) or low (≤ median) frequency of tumor antigen reactive, KIR^+^CD8^+^ T cells identified using the Lasso Logistic model classifier in patients with advanced melanoma (n = 29) and C) the patients previously resistant to immunotherapy (n = 12). D) Three-year overall survival based on a high (> median) or low (< median) frequency of the tumor antigen reactive, KIR^+^CD8^+^ T cells in the flow cytometry cohort (n = 47). Between group differences are measured by Gehan-Breslow-Wilcoxon test (*p* < 0.05).

To assess the potential clinical relevance of KIR^+^CD8^+^ T cells, we examined overall survival and its association with the frequency of KIR^+^CD8^+^ T cells in our single-cell sequencing cohort. We adopted a Lasso Logistic model to construct a classifier to prospectively classify cells based on clinical variables and select gene expression. We focused on genes encoding cell surface proteins where gene-level expression positively correlated with protein-level expression based on three blood samples for which we performed paired single-cell RNA and DNA oligonucleotide antibody labeling (**table S1, S9**), which may allow for future validation using clinically-available techniques, such as flow cytometry (**fig. S8**). Training and testing datasets were generated from the immunotherapy-naive single-cell RNA sequencing cohort, which demonstrated a high prediction performance (Training: AUC 0.864, Testing: AUC 0.867; Fig. 5A). Application to patients who had disease progression following immunotherapy (immunotherapy resistant cohort) also demonstrated acceptable prediction performance (AUC 0.753), suggesting the potential generalizability of the model regardless of past treatment history. We then applied this model to the combined single-cell sequencing cohorts. Using a median cut-point to define patients with high (> median) or low (≤ median) frequencies of predicted-reactive KIR^+^CD8^+^ T cells, we observed that patients with a higher proportion of predicted-reactive KIR^+^CD8^+^ T cells in the blood had shorter three-year overall survival (*p* = 0.037; Fig. 5B, **table S11**). This inverse association was particularly pronounced in the immunotherapy resistant cohort (*p* = 0.029; Fig. 5C, **table S11**).

To validate the association of KIR^+^CD8^+^ T cells and worse overall survival, we next examined the prognostic significance of KIR^+^CD8^+^ T cells in our flow cytometry (n = 47, table S10). Using a median cut-point to define patients with high versus low frequencies, we again observed that patients with a higher proportion of KIR^+^CD8^+^ T cells in the blood had shorter three-year overall survival (*p* = 0.047; Fig. 5D, **table S10**). Overall, the association with worse clinical outcome is consistent with the potential regulatory nature of KIR^+^CD8^+^ T cells on anti-tumor immunity.

## DISCUSSION

In this study, we employed high-resolution single-cell techniques to profile tumor antigen-specific T cells in patients with advanced melanoma. By using TCR sequences as a molecular barcode to link clonally related T cells in the tumor and blood, we identified a subpopulation of circulating predicted-reactive CD8^+^ T cells resembling KIR^+^CD8^+^ Tregs, which have been shown to specifically kill pro-inflammatory, autoreactive T cells in patients with autoimmune diseases and viral infections. Using an unbiased epitope screen, we experimentally confirmed the tumor antigen specificity of KIR^+^CD8^+^ T cells. To our knowledge, our study is the first to demonstrate the tumor antigen specificity of KIR^+^CD8^+^ Tregs in patients with cancer.

While counterintuitive in the context of anti-tumor immunity, as tumor antigen-specific CD8^+^ T cells are the predominant effector immune cells necessary for tumor clearance, it is possible that the suppressive function in KIR^+^CD8^+^ Tregs may represent a differentiation state induced by tumor cells as a mechanism of immune evasion. Similar mechanisms have been described with CD4^+^ Tregs, whereby tumor cells induce the proliferation of tumor antigen-specific, suppressive CD4^+^ Tregs through the direct and indirect presentation of human leukocyte antigen (HLA) class II tumor antigens^6^. An analogous induction of CD8^+^ Tregs through the presentation of HLA class I tumor antigens, as observed in our data, is possible, and prior *ex vivo* studies suggest that TCR signaling in KIR^+^CD8^+^ Tregs is required for maximal suppressive function^2^. A recent study additionally described the peripheral induction of Ly49^+^CD8^+^ Tregs from thymic extruded T cells after encountering self-antigen in mouse models, which impaired anti-tumor immunity^36^, lending further support for the antigen-specific nature of CD8^+^ Tregs.

Our analyses also link KIR^+^CD8^+^ T cells in the tumor and blood and demonstrate the potential relevance as a clinical, blood-based biomarker. Importantly, we observed that the expression of a conserved gene signature in KIR^+^CD8^+^ T cells is correlated between tumor and blood, providing rationale for using the blood to detect the presence of KIR^+^CD8^+^ T cells in the tumor. Lastly, KIR^+^CD8^+^ T cells are associated with worse overall survival in patients with advanced melanoma, which is consistent with the immunosuppressive function of CD8^+^ Tregs.

Our study has several limitations. While our epitope screen and analyses mapping clonotypes to publicly available references were able to identify tumor antigen specificity in a subset of KIR^+^CD8^+^ T cells, the epitope specificity of the remaining of KIR^+^CD8^+^ T cells is unknown. As KIR^+^CD8^+^ T cells have been described at homeostasis and increase with progressive inflammation as an alternative mechanism to preserve peripheral tolerance, it is unclear whether tumor antigen specificity is required to impair anti-tumor immunity or whether the presence of KIR^+^CD8^+^ T cells is instead representative of host immunity more broadly. Further studies are also required to elucidate the specific interactions and contexts needed to induce KIR^+^CD8^+^ Tregs. Additionally, the specific mechanism by which KIR^+^CD8^+^ Tregs impair anti-tumor immunity remain unclear. While KIR^+^CD8^+^ Tregs have been described to target antigen-specific CD4^+^ T cells through interactions between both classical and non-classical major histocompatibility complex interactions in autoimmunity^2,19^ and one prior study reported that the interactions between FOXP3^−^CD8^+^CD25^+^KIR^+^CD127– and CD4^+^ Tregs were important in influencing anti-tumor immunity^3^, we observed no association between the frequency of KIR^+^CD8^+^ T cells and CD4^+^ T cell subsets in the tumor microenvironment (data not shown). We did observe a trend towards an inverse correlation between the frequency of KIR^+^CD8^+^ T cells and other neoTCR8 predicted-reactive CD8^+^ T cells in the tumor, raising the possibility that KIR^+^CD8^+^ Tregs may target other neoantigen-specific CD8^+^ T cells.

Overall, our study demonstrates the potential clinical relevance of KIR^+^CD8^+^ Tregs in patients with advanced melanoma. Importantly, the presence of KIR^+^CD8^+^ Tregs in the blood was indicative of the tumor immune microenvironment, providing rationale for further exploration of this population as a circulating biomarker and potential therapeutic target. The prognostic significance of KIR^+^CD8^+^ Tregs regardless of treatment history further raises the possibility that this population is not impacted by current immune checkpoint inhibitor therapies and may be a relevant mechanism of therapeutic resistance. Further insight into the function of KIR^+^CD8^+^ Tregs in the tumor microenvironment may lead to the development of future therapies.

## Methods

### Patient samples

Freshly resected, histologically-confirmed tumor tissue and/or blood were collected from patients with cutaneous melanoma. Tumor samples were obtained immediately following surgery and processed, as outlined below. Heparinized blood samples were collected prior to surgery or treatment and immediately processed, as outlined below. Patient sample details, including the tissue site sampled, processing details, sex, age, treatment history, and clinical outcome, are available in **table S1**.

### Tumor dissociation and peripheral mononuclear cell isolation

Single-cell tumor and blood suspensions were prepared, as previously described^7^. In brief, freshly resected tumor samples were mechanically dissociated and digested in HBSS medium with collagenase IV (2.5 mg/ml; Roche) and DNase I (0.2 mg/ml; Worthington Biochemical Corporation) at 37°C for 30 min. Dissociated tumor suspensions were then isolated using Lymphoprep gradient centrifugation. Peripheral blood mononuclear cells were isolated from whole blood using Lymphoprep gradient centrifugation. For samples Mel-T-09, Mel-T-11, Mel-UT-03, Mel-UT-05, Mel-UT-08–13, and Mel-UT-17–18, single-cell suspensions were cryopreserved in GemCell human AB serum (Gemini) with 20% DMSO in liquid nitrogen. The single-cell suspensions were then thawed per 10X Genomics protocols CG000233 and CG000447 for tumor and blood samples, respectively, prior to further processing.

### Cell sorting

Single-cell suspensions were stained for live cells (Live/Dead Cell Viability Assay; Life Technologies) followed by fluorophore-conjugated anti-human antibodies, as summarized in **table S2**. Samples were sorted on a BD FACS Aria II with the gating strategies outlined in **table S3**. At least 30,000 live cells were sorted per sample.

### CITEseq antibody staining

Cryopreserved peripheral blood mononuclear cell suspensions were thawed, as above. Lyophilized TotalSeq^™^-C Human Universal Cocktail V1. 0 (BioLegend) were reconstituted and used for staining, per the manufacturer’s protocols. Each sample was reconstituted to a concentration of 20 million cells/mL and incubated with Human TruStain FcX^™^ Fc Blocking reagent (BioLegend) for 10 minutes at 4°C. The blocked cells were then stained with the reconstituted TotalSeq-C antibody cocktail and fluorophore conjugated surface antibodies for cell staining (**table S2**) for 30 minutes at 4°C as washed with Cell Staining Buffer (BioLegend) prior to cell sorting (**table S3**).

### 10X library preparation and sequencing

Sorted samples were prepared for single-cell sequencing by the Keck Microarray Shared Resource (KMSR) and sequenced by the Yale Center for Genome Analysis (YCGA) at Yale University. Cells were processed following the recommended protocol with the Chromium Single Cell 5’ Library Construction Kit and Chromium Single Cell V(D)J Enrichment Kit (Human T Cell; Single Cell 5’ Chemistry). Libraries were sequenced on an Illumina NovaSeq S4 instrument. scRNAseq libraries were sequenced at a read length of 26 × 8 × 91 bp and a depth of 300 million reads per sample. CITEseq libraries were sequenced at a read length of 26 × 8 × 91 bp and a depth of 100 million reads per sample. scTCRseq libraries were sequenced at a read length of 150 × 150 bp and a depth of 20 million reads per sample. FASTQ files were generated and analyzed with CellRanger (version 5.0.1) using the GRCh38 human reference genome for alignment.

### Whole exome sequencing

Melanoma tumors and adjacent normal tissue were analyzed with whole-exome sequencing, as previously described in-detail^37^. Briefly, genomic DNA was extracted from snap-frozen tumors using DNeasy purification kits (Qiagen Inc.). Genomic DNA was sheared, end repaired, ligated with custom adapters (Integrated DNA Technologies), amplified, and size selected. Sample concentrations were normalized to 2nM and sequenced on an Illumina NovaSeq 6000 using 101 bp paired-end sequencing reads, per Illumina protocols. Raw sequencing files were aligned to the GRCh38 human genome.

### Bulk RNA sequencing

Bulk RNA sequencing was performed on melanoma tumors, as previously described^37,38^. In brief, RNA was extracted from tumors using the RNeasy PowerLyzer Tissue & Cells Kit (QIAGEN) and quality was assessed with a 2100 Bioanalyzer System (Agilent Technologies). Ribosomal RNA was depleted Kapa RNA HyperPrep Kit with RiboErase (Kapa Biosystems, Inc., Cape Town, South Africa). Sequencing libraries were generated using TruSeq RNA sample prep kits (Illumina) and sequenced on an Illumina HiSeq 2500 using 2 × 150 bp paired-end reads with a target of 20–25 million reads per sample. Raw sequencing files were aligned to the GRCh38 human genome.

### Immunophenotyping human peripheral blood mononuclear cells

Cryopreserved peripheral blood mononuclear cells were thawed, as above, and rested at 37°C for 16 hours. Cells were then stained for viability with Fixable Near-IR Dead Cell Stain (Life Technologies) at room temperature for 10 minutes in the dark. Samples were then washed with PBS with 2% FBS, prior to staining with a master mix of surface antibodies (**table S2**) at room temperature for 20 minutes. Cells were then washed with PBS with 2% FBS. Samples undergoing intracellular staining were fixed for 45 minutes with Fixation Buffer, per the manufacturer’s protocol (eBioscience). Cells were washed with Permeabilization Buffer (eBioscience), stained with a master mix of intracellular antibodies (**table S2**) at 4°C for 16 hours, and resuspended in PBS + 10% FBS + 0.2% EDTA for analysis on a BD LSR Fortessa in the Yale Flow Cytometry Core.

### MCR construction

HLA typing was computationally inferred from the single-cell RNA sequencing data from blood cells using the python-based package, arcasHLA^39^. MCR libraries were generated by cloning oligonucleotide libraries (Twist Bioscience) into MCR-SCTz retroviral expression vectors (having an IRES-hCD4tr (1–420aa) downstream) for each patient HLA, as described previously^30^. For cloning of single peptide-MCR constructs, oligos were purchased from Microsynth AG (Balgach, Switzerland). The libraries cover the full length of selected antigens, as detailed below, by sliding 10-mers shifted by one amino acid. After retroviral production, 16.2X cells were transduced with the MCR libraries. After 2–3 days, transduced cells were sorted based on hCD4 expression (BioLegend) on a BD FACSAria Fusion.

### TCR cloning

Retroviral expression vectors with TCRs as a bicistronic TCRα-T2A-TCRβ transcript were produced by Twist Bioscience. 16.2A2 cells (16.2 T cell hybridoma expressing human CD3E, CD3G, and CD3D) were transduced and after 2–3 days, TCR expressing (BioLegend) cells sorted on a BD FACSAria Fusion.

### MCR screening

MCR screening was performed as previously described^29,30^, with the adaption of pooling of up to five different TCRs per co-culture. TCR-cells and MCR-cells were co-cultured with a 5-fold excess of each TCR compared to MCR-cells. Up to 7 rounds of enrichment were done. When a NFAT-signal was detected, the enriched MCR cells were co-cultured with the individual TCR-expressing cell lines to identify the TCR recognizing the MCR cells. Sorting was performed on BD FACSAria Fusions, flow-cytometry for acquisition only was done on a BD LSRFortessa or Luminex Guava easyCyte. When multiple peptides (due to multiple transduction) were detected in the sequencing of positive single cell clones, the peptides were subcloned and re-expressed as single peptides-MCR-SCTz (e.g. TCR2328). Expanded single cells were harvested, and DNA was isolated (KAPA Express Extract), followed by Sanger sequencing of the linked peptide and the HLA class I heavy chain.

### Data Processing and Statistics

#### Single-cell RNA sequencing processing and analysis

CellRanger output matrices containing gene expression values for each cell were analyzed using the open-source toolkit, Seurat v4.2^40^. Cell level quality control was performed for each sample separately. Thresholds for removing low-quality cells were selected based on the distribution of the number of unique genes per cell, the number of genes per unique molecular barcode (novelty score), the percentage of genes that map to the mitochondrial genome, and the expression of housekeeping genes. Library size normalization and variance stabilization was performed using SCTransform to apply a regularized negative binomial regression based on the 3,000 most variable genes^41^. Genes encoded by the Y chromosome were removed for downstream analysis. Normalized datasets from tumor and blood samples for each patient were then combined using the FindIntegrationAnchors and IntegrateData functions in Seurat with default parameters^42^. Scaled z-scores for each gene were calculated and used for principal component analysis (PCA) based on the integrated dataset.

Cell clustering was performed by first using the shared nearest neighbor (SNN) method based on statistically significant principal components, as applied by the FindNeighbors function. Differentially expressed genes between clusters of interest were then identified using the Wilcoxon Rank Sum test with Bonferroni correction (*q* < 0.05)^43^. For subsequent T cell subset analyses, T cell receptor genes were removed to mitigate the effects of clustering driven strictly by highly expanded clones. Cluster annotation was manually assigned based on the expression of canonical marker genes, differentially expressed genes, overlap coefficients with previously published cell phenotype gene signatures^5,25,44,45^, automated reference-based annotations^46^, and the proportion of cells with paired T cell receptor CDR3α and CDR3β chains. Clusters with T cells were selected for downstream T cell phenotyping (**table S4-S5**). *CD8A, CD8B, and CD4* expression following Adaptively-thresholded Low Rank Approximation imputation (ALRA, version 1.0.0)^47^ were also used to assign CD8^+^ and CD4^+^ phenotypes.

#### T cell receptor repertoire analysis

T cell receptor contigs called with high confidence were extracted from CellRanger VDJ outputs and retained for downstream analyses. Nucleotide and amino acid CDR3α and CDR3β sequences and count matrices of gene expression were matched for each cell based on barcode identities using custom R scripts. Only cells with a single CDR3α and single CDR3β sequence were retained for downstream analyses. Cells with multiple sequences or those with missing sequences were not considered for clonal analyses. Clonal relatedness was defined by cells with exact matching single CDR3α and single CDR3β sequence per patient.

To quantify the degree of clonal expansion, we calculated the proportion of T cell clones occupied by a given clonotype per patient. To predict T cell receptor specificities, we matched clonotype sequences in our dataset with VDJdb, a publicly available database of T cell receptor sequences annotated with experimentally validated specificities^13,48^. Human species clonotypes were extracted from VDJdb and matched based on exact amino acid sequences of the CDR3 from the paired a-chain and b-chains. We then calculated the number of tumor antigen-specific and viral-specific clonotypes in each reactivity group.

Additional single-cell T cell receptor repertoire analysis was performed using the scRepertoire package (version 1.12.0)^49^. Filtered annotated contig files generated by the CellRanger VDJ pipeline were used as input files. Cells with paired single alpha- and beta-chains were selected for downstream analysis. Clonotypes were defined by using the CDR3 nucleotide and amino acid sequences. Indices of diversity (Shannon entropy, Inverted Simpson) and species richness (Chao, ACE) were calculated using clonalDiversity function. Between group differences were calculated with Kruskal-Wallis one-way analysis of variance testing and Dunn’s multiple comparison testing. Visualization of repertoire metrics were generated using GraphPad Prism (version 10.1.1).

#### Differential abundance analysis

To unbiasedly identify cellular neighborhoods enriched for tumor matched reactive or unreactive circulating CD8^+^ T cells, we used an R implementation of Local Two-Sample Testing for differential abundance protocols^15^. The first 30 dimensions of principle components and k = 25 were used to construct a k-nearest neighbors (KNN) graph of the data using Euclidian distances. Random walk scan statistics were then calculated (*q* < 0.05), and differential abundance labels were assigned if *q* < 0.01. Differentially abundant cells were then grouped into different clusters by employing Seurat SNN clustering with default parameters. All differentially expressed genes were then calculated for each differentially abundant cluster using the FindAllMarkers function in Seurat (**table S6**).

#### Single-cell gene set enrichment analyses

Gene set enrichment analysis of the neoTCR scores, the KIR^+^CD8^+^ signature, and the conserved KIR^+^CD8^+^ signatures were calculated from the cell counts of the single-cell RNA sequencing data using the R-based package AUCell (version 1.24.0)^50^. Gene sets were considered active based on the internal global_k1 threshold or through a manual examination of the distribution pattern.

Single-cell gene signature scoring was performed using the R-based package UCell (version 2.1.2) with default parameters^51^. Between group differences were calculated using Wilcoxon Rank Sum testing with continuity correction (*q* < 0.05).

Gene set enrichment analyses between gene lists was performed using the GSEA software (version 4.3.2)^52^ Differentially expressed genes from differentially abundant populations were pre-ranked based on the fold-change calculated from Wilcoxon signed-rank testing with Bonferroni correction (*q* < 0.05). Pre-ranked gene lists were then compared with the top 200 genes upregulated in human KIR^+^CD8^+^ T cells from patients with multiple sclerosis^2^ and immune signatures from Hallmark gene sets in the Molecular Signatures Database (MSigDB)^53^.

#### Conserved gene signature analysis

Differentially upregulated genes shared between circulating KIR^+^CD8^+^ T cells and clonally related cells in the tumor were identified using the FindConservedMarkers function in Seurat. To derive the conserved signature, differentially expressed genes were first identified between KIR^+^CD8^+^ T cells and all other CD8^+^ T cells in the blood and tumor separately based on Wilcoxon Rank Sum testing with Bonferroni testing for multiple-comparison correction. Differentially expressed genes across tissue compartments (maximum *p* < 0.01), expression in ≥1% of cells, and an average log-fold change ≥ 0.15 were considered conserved and used for downstream analysis (**table S7**).

#### Trajectory analyses

To perform trajectory inference analysis, we fit a minimum spanning spanning tree (MST) to the UMAP dimensionality reduction plot using the Slingshot package (version 2.7.0)^54^. The piecewise linear trajectory was smoothed using simultaneous principal curves to derive trajectory and pseudotime values. To determine associations between gene expression and pseudotime values, we fit a negative binomial general additive model using the tradeSeq package (version 1.10.0)^55^. Pairwise differences in the gene expression pattern between lineages was determined using the Wald test (*p* < 0.05), as applied by the patternTest function with default parameters (**table S8**).

#### Neoantigen prediction

Aligned sequencing reads generated by whole exome sequencing were analyzed for somatic variants by NEXUS Personalized Health Technologies core at ETH Zurich using the SwissMTB workflow^56^. Somatic mutations were selected if identified by at least two of three variant callers (Strelka2, MuTect2^57^, VarScan^58^). Somatic variants were subsequently annotated using Ensembl Variant Effect Predictor^59^.

#### Antigen selection for MCR library

Peptides derived from neoantigens identified above and shared antigens were included for MCR screening. Shared antigens included curated tumor associated antigens, select human endogenous retroviruses (HERVs), previously described driver mutations^60^, and previously described bacterial peptides from melanoma tumors^61^. Sequences from cytomegalovirus, Epstein Barr virus, and influenza peptides from the Immune Epitope Database were included as controls^62^.

Tumor associated antigens were first selected by performing differential expression analysis between 369 metastatic melanoma samples from The Cancer Genome Atlas (TCGA) and 974 normal skin controls from the Genotype-Tissue Expression (GTEx)^63^. Genes were filtered for expression of ≥10 transcripts per million (TPM), ≥10% of samples, and ≥3-fold change expression in melanoma tumors were selected. Identified genes were further filtered for a median expression ≥ 5 TPM in the bulk RNA sequencing data generated from 20 tumors from 13 patients in the Yale cohort (**table S1**), yielding 66 total genes. An additional 29 genes from known and investigational tumor associated antigens were also included for MCR screening.

Human endogenous retroviruses were selected by first aligning raw reads from the bulk RNA sequencing data from the melanoma tumor in the Yale cohort to a previously published reference of pro-viral sequences^64^. HERVs pro-viral sequences with expression levels ≥10 TPM in ≥3 tumors and with previously annotated gene identifiers were selected, resulting in 19 genes. An additional 5 genes associated with HERV-driven oncogenesis or eliciting T cell responses were also included.

#### CITE-seq data processing and analysis

Raw and filtered UMI matrices for antibody-derived tag (ADT) counts generated from the CellRanger pipeline were normalized with the dsb package (version 1.0.3) using default parameters^65^. Background droplets were identified by examining the protein library size distribution. The normalized ADT matrices were then added to Seurat objects generated from the RNA count matrices and analyzed, as described above.

#### Surface protein-encoding gene selection

To identify candidate surface markers where gene expression correlated with protein-level expression, we calculated Spearman’s Rank correlation coefficients between normalized expression in the ADT and RNA assays. Of 137 markers analyzed, 40 genes positively correlated (r ≥ 0.1) with protein-level expression and were therefore included in the surface marker classifier models (**table S9**).

#### Cell classification analysis

To further investigate the relationship between surface markers and tumor reactivity, we adopted a Lasso Logistic model^66–68^ to analyze normalized count matrices from CD8^+^ T cells (glmnet version 4.1–4). Training and testing datasets were constructed by randomly splitting cells from the immunotherapy-naive cohort into equal sample sizes. In the training phase, the Lasso Logistic model was fitted to the count matrices for the 40-surface protein-encoded genes, select clinical variables (sex, age, clinical stage, lesion location, technical batch), and predicted reactivity. The classifier was then applied to the testing datasets and the immunotherapy resistant cohorts. Receiving operating characteristics statistics were calculated based on the differential abundance reactivity labels for the immunotherapy naive cohort and neoTCR8 reactivity labels for the immunotherapy resistant cohort and visualized using the R-based package pROC (version 1.18.0).

To facilitate the application of the Lasso Logistic model classifier to clinically applicable techniques such as flow cytometry, we adopted a Decision Tree model to assign hierarchical combinations of markers using samples where simultaneous RNA sequencing and CITE-seq were performed. Testing and training datasets were constructed by randomly splitting cells from the CITEseq cohort. Thresholds to dichotomize protein-level expression of the markers selected by the Lasso Logistic model classifier were determined by examining the pattern of expression in the ADT assay. A Decision Tree was constructed based on the binary protein expression levels to predict tumor reactivity in the training set using R-based package rpart (version 4.1.19).

#### Flow cytometry data analysis

Flow data was analyzed using FlowJo^™^ (version 10.9). Gating was determined by comparison to Fluorescence Minus One (FMO) controls using healthy donor human peripheral blood mononuclear cells.

#### Survival analysis

The association between the frequency of reactive KIR^+^CD8^+^ T cells (high, or greater than or equal to the median frequency for that cohort, vs. low, or less than the median frequency for the cohort) as classified by flow cytometry staining (**table S10**), the Lasso logistic regression classifier (**table S11**), or the decision tree model and overall survival (OS) time was assessed by Kaplan-Meier analysis. We used the Gehan-Breslow-Wilcoxon test for trend (*p* < 0.05) to detect significant differences in OS between the high proportion and low proportion groups. All survival analyses were performed using GraphPad Prism (version 10.1.1).

#### Study approval

This study was approved by the Yale University Institutional Review Board. All participants provided written, informed consent before tissue and/or blood collection.

## Data Availability

Further information and requests for resources and reagents should be directed to and will be fulfilled by the corresponding authors.

## Data and materials availability:

Raw sequencing data files will be available through the National Center for Biotechnology Information (NCBI) dbGaP at accession no. phs002289.v2. All biological materials and computer code used in this study are available from the corresponding authors upon reasonable request.
